# DNA repair deficiency in peripheral blood lymphocytes of endometrial cancer patients with a family history of cancer

**DOI:** 10.1186/1471-2407-14-765

**Published:** 2014-10-15

**Authors:** Lyubov Buchynska, Olga Brieieva, Nadiia Glushchenko, Ludmila Vorobyova, Olena Bilyk

**Affiliations:** R.E. Kavetsky Institute of Experimental Pathology, Oncology and Radiobiology, NAS of Ukraine, Kyiv, Ukraine; National Cancer Institute, Kyiv, Ukraine

**Keywords:** DNA damage, Bleomycin, DNA repair capacity, Endometrial cancer, Family history of cancer, Comet assay

## Abstract

**Background:**

Individual susceptibility to endogenous and/or exogenous DNA damage depends on DNA repair efficiency and can be evaluated using the comet assay with bleomycin as genotoxic agent. The aim of the study was to evaluate baseline and bleomycin-induced DNA damage and DNA repair capacity in peripheral blood lymphocytes (PBLs) of endometrial cancer (EC) patients considering a family history of cancer.

**Methods:**

DNA damage was analyzed in PBLs of 45 EC patients compared to a control group of 10 healthy women, using the comet assay. The level of DNA damage was determined by the% tail DNA.

**Results:**

The level of baseline DNA damage in PBLs of EC patients was significantly higher (% DNA in tail 9.31 ± 15.32) than in healthy women (% DNA in tail 3.41 ± 4.71) (P <0.01). PBLs of EC patients repaired less bleomycin-induced DNA damage (removed% DNA in tail 63.94 ± 20.92) than PBLs of healthy individuals (removed% DNA in tail 80.24 ± 3.03) (P <0.001). Efficiency of DNA repair in PBLs of EC patients depended on the family history of cancer. The amount of restored damaged DNA was significantly lower (removed% DNA in tail 36.24 ± 14.05%) in EC patients with a family history of cancer compared to patients with sporadic EC (removed% DNA in tail 64.91 ± 19.36%) (P <0.004).

**Conclusions:**

Lymphocytes of EC patients are characterized by an increased basal level of DNA damage as well as deficiency in DNA repair. DNA repair is less efficient in PBLs of EC patients with a family history of cancer compared to patients with sporadic cancer.

## Background

The cell genome is constantly exposed to endogenous and/or exogenous genotoxic agents causing structural DNA changes which have potentially tremendous cellular consequences. The ability of a cell to decrease or eliminate entirely DNA damage depends on a variety of factors including the nature of the DNA damage and efficiency of DNA repair. A deficiency in DNA repair capacity leads to greater DNA damage mediated by genotoxic agents and may result in accumulated DNA damage. Both these factors exponentially contribute to increasing the risk for cancer through genomic instability [1–3].

Human cancers have been shown to demonstrate greater genomic instability compared with the normal tissue in the same host. However, several studies report genomic instability not only in cancer but also in somatic non-cancer cells of cancer patients, particularly in peripheral blood lymphocytes (PBLs) [4–11]. These studies hypothesize that the level of genome damage in PBLs suggests an individual susceptibility to cancer [12–14].

To date, little is known about spontaneous genome damage in PBLs of cancer patients with a family history of cancer. Smith et al. did not find any correlation between DNA damage in PBLs of breast cancer patients and a family history of cancer [12]. In contrast, Roy et al. [13] have demonstrated that PBLs of breast cancer patients and their healthy blood relatives with a family history of cancer were highly sensitive to genotoxic effect of bleomycin as compared to controls. Moreover, an increased level of chromosomal aberrations was found in PBLs of colorectal cancer patients with a family history of cancer [14].

It is well known that endometrial cancer (EC) may arise within hereditary cancer syndromes (Lynch syndrome, Li-Fraumeni syndrome, etc.). In addition, patients with EC may have a family history of malignant tumors of organs of the female reproductive system, gastrointestinal tract and some others associated to Lynch syndrome [15–17]. One study has reported the presence of an increased frequency of chromosomal aberrations and breaks at fragile sites of chromosomes in PBLs from EC patients with a family history of cancer compared to PBLs from EC patients without such a family history [18]. The observed chromosomal instability in PBLs may be caused either by inherited susceptibility to genotoxic exposure or by low ability to repair DNA lesions. Therefore, a study to evaluate the sensitivity of PBLs to genotoxic mutagens and the DNA repair capacity in EC patients with a family history of cancer is important. Such approach could contribute to an efficient selection of family members at high risk for cancer and help design cancer prevention strategies for individuals at risk.

The aim of the study was to analyze the level of DNA damage and DNA repair efficiency in peripheral blood lymphocytes of EC patients, taking into account whether or not they had a family history of cancer.

## Methods

Forty five patients with endometrial cancer (EC) stage I and II and ten healthy women were included in the study after having obtained signed informed consent. The number of participants had sufficient statistical power to obtain statistically significant results. EC patients underwent surgical treatment at the gynecological oncology department of the National Cancer Institute, Kyiv, Ukraine. All studied tumors were classified as endometrioid adenocarcinomas. The mean age of the EC patients was 60 years, while that of healthy individuals was 55.7 years. The study was approved by the Committee for Ethical Issues of the R.E. Kavetsky Institute of experimental pathology, oncology and radiobiology, National Academy of Sciences of Ukraine.

A standardized questionnaire was used to collect information on family history of cancer, age, work environment, medications, smoking status, alcohol consumption and nutritional factors. Only EC patients with approximately the same lifestyle were included in this study. We selected 12 of the 40 (30%) of the EC patients with a strong family history of cancers of female reproductive organs (endometrium and ovary), gastrointestinal tract and some other cancers associated to Lynch syndrome [15–17].

Venous blood samples were obtained by venipuncture and collected into EDTA tubes before patients had received any chemotherapy or radiation therapy. Lymphocytes from whole blood were isolated by centrifugation through Ficoll-Hypaque.

Analysis of baseline and bleomycin-induced DNA damage was performed on freshly isolated PBLs using an alkaline comet assay [19, 20]. Briefly, microscope slides were first covered with a 1% normal melting point agarose. Then, 1 – 2 × 10^5^ of cells were embedded into 75 μL of 1% low-melting point agarose at 37°C and the gel was cast over the first agarose layer. To study genotoxic DNA damage, lymphocytes were treated with bleomycin at concentration 20 μg/ml in phosphate buffered saline (PBS), рН 7.4, for 30 min, as recommended by Schmezer et al. [20]. DNA repair capacity was evaluated as the extent of removal of damage in lymphocytes after immersion slides into PBS without bleomycin and incubation for 15 min, 37°C. As a positive control, lymphocytes treated with 100 μM Н_2_О_2_ for 5 min were used. Then slides were immersed into a lysis solution (2.5 M NaCl, 100 mM EDTA, 10 mM Tris, 10% DMSO, 1% Triton X-100, pH10) and kept for an hour at 4°C. After cell lysis, slides were placed in a horizontal gel electrophoresis unit filled with alkaline electrophoresis buffer (300 mM NaOH, 1 mM EDTA, pH13). After 20 min of alkali treatment, electrophoresis was performed for 20 min at 0.8 V/cm. Slides were then neutralized 2×10 min using neutralization buffer (0.4 M Tris, pH 7.5) and stained with SYBR Green I. Comets were analyzed using the computer-based image analysis system CometScore (TriTek Corp., Sumerduck, VA, USA). The level of DNA damage was expressed as% DNA in tail.

Data were analyzed using Statistica 8.0 (StatSoft, Inc.) software. To compare differences between groups, the Mann-Whitney nonparametric test was used with a significance level of P <0.05.

## Results

**Figure 1:**
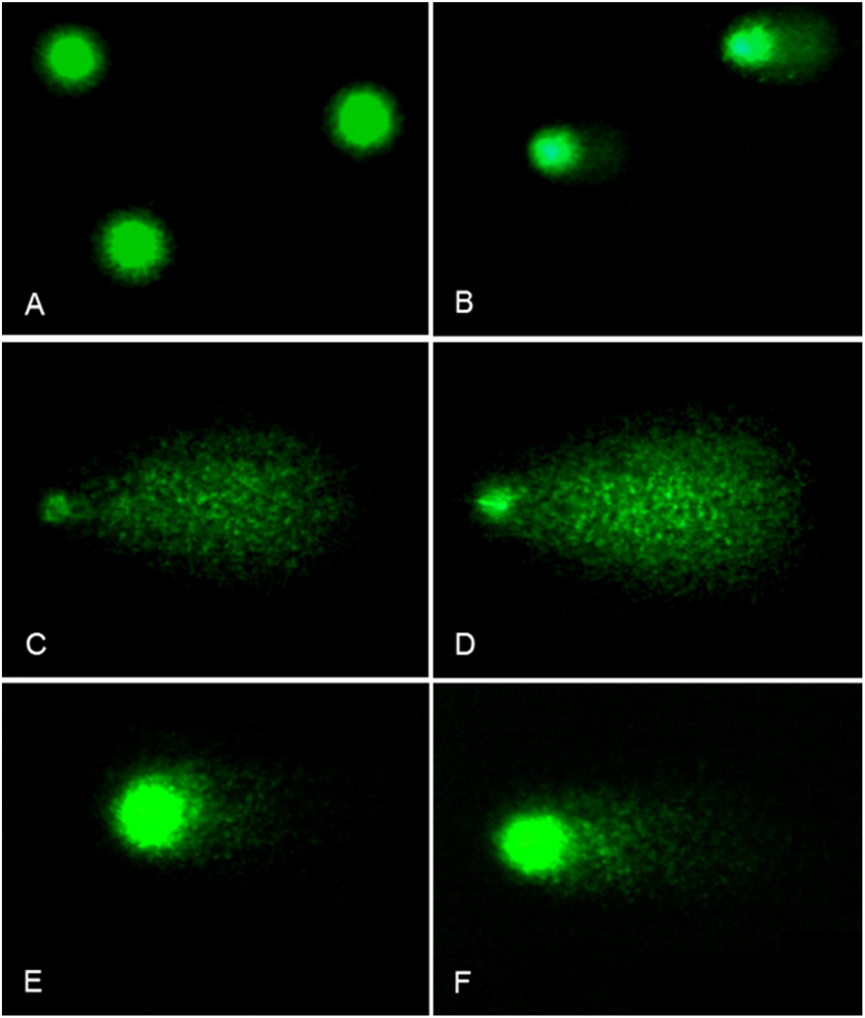
**Examples of DNA comets obtained by comet assay in peripheral blood lymphocytes of healthy individuals (A,C,E) and EC patients (B, D, F). A**, **B**. Baseline DNA damages; **C**, **D**. DNA damage after bleomycin exposure; **E**, **F**. DNA damage remained after repair.

**Figure 2:**
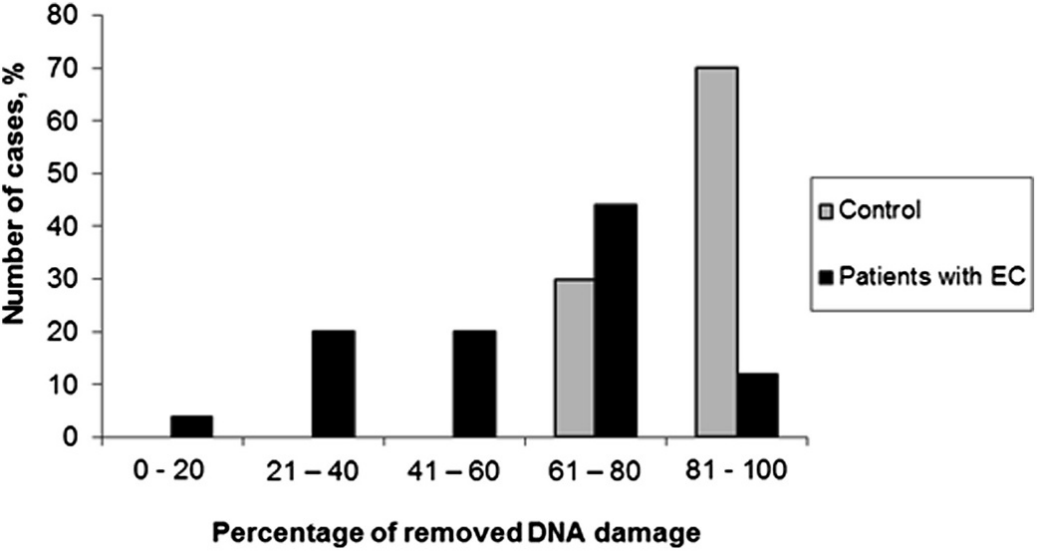
**Bleomycin-induced DNA damage removed in PBLs of healthy individuals and EC patients.**

We observed a significantly (P <0.01) higher baseline DNA damage in EC patients (% DNA in tail 9.31 ± 15.32) as compared with healthy individuals (% DNA in tail 3.41 ± 4.71) (Table 1, Figure 1). The net bleomycin-induced DNA damage was assessed by subtracting the basal% DNA in tail from the% DNA in tail obtained after incubating the PBLs with bleomycin. It was observed that it didn’t differ significantly in EC patients (% DNA in tail 89.35 ± 3.99) compared to healthy individuals (% DNA in tail 84.20 ± 5.38). However, we found differences after we removed bleomycin and studied DNA damage repair. The level of DNA damage in PBLs decreased within the first 15 min after removal of bleomycin, both in healthy individuals and in EC patients (Table 1, Figure 1). However, it was observed that the level of DNA damage restored in PBLs of EC patients was significantly (P <0.001) lower (removed% DNA in tail 63.94 ± 20.92) than in PBLs of control individuals (removed% DNA in tail 80.24 ± 3.03). Most importantly, we noted 80 - 100% recovery of bleomycin-induced DNA damage in 70% of healthy individuals and in only 12% of EC patients (Figure 2).

**Table 1:** **DNA damage in peripheral blood lymphocytes of EC patients**

Groups of examined patients (n =55)	% tail DNA mean ± SD		
	Baseline DNA damage	Net bleomycin-induced DNA damage	Removed DNA damage
Healthy individuals (n =10)	3.41 ± 4.71	84.20 ± 5.38	80.24 ± 3.03
EC patients (n =45)	9.31 ± 15.32*	89.35 ± 3.99	63.94 ± 20.92*

*Difference is significant in respect to the control group (р < 0.05).

To determine the significance of hereditary factor for genetic instability in PBLs, we studied the level of baseline DNA damage, bleomycin sensitivity and DNA repair capacity in EC patients in relation to a family history of cancer. We did not observe any significant increase in background DNA damage in PBLs of EC patients with a family history of cancer (% DNA in tail 10.35 ± 16.35) compared to those with no family history of cancer (% DNA in tail 9.51 ± 15.71) (Table 2). The net bleomycin-induced DNA damage also did not differ significantly between the two groups (% tail DNA 91.09 ± 2.52 and 88.56 ± 4.25 respectively). However, significant difference (P <0.004) in repair efficiency was found among these patients. By further incubating cells in PBS without bleomycin, PBLs of EC patients with a family history of cancer were able to repair less bleomycin-induced DNA damage (removed% tail DNA 36,24 ± 14,05) compared to patients with no family history of cancer (removed% tail DNA 64,91 ± 19,36) (Table 2).

**Table 2:** **DNA damage in peripheral blood lymphocytes of EC patients related to a family history of cancer**

Groups of examined patients (n =40)	% tail DNA mean ± SD		
	Baseline DNA damage	Net bleomycin-induced DNA damage	Removed DNA damage
Patients with a family history of cancer (n =12)	10.35 ± 16.35	91.09 ± 2.52	36.24 ± 14.05
Patients with no family history of cancer (n =28)	9.51 ± 15.71	88.56 ± 4.25	64.91 ± 19.36*

*Difference is significant in respect to the group of patients with a family history of cancer (р < 0.05).

## Discussion

The results of our study indicate that lymphocytes of EC patients are characterized by pronounced genome instability resulting in increased basal level of DNA damage and deficiency in DNA repair. However one of the limitations of this study was the small sample size we did observed significantly higher baseline DNA damage in EC patients than in healthy individuals which is in agreement with other studies using the comet assay as the study endpoint. Higher baseline DNA damage in lymphocytes was observed in breast cancer patients in comparison with healthy volunteers [11, 12]. Kurzawa-Zegota et al. showed that lymphocytes from colon cancer patients had greater baseline DNA damage than those from healthy individuals and this higher level of damage was also observed throughout in vitro treatment with genotoxins [21]. Moreover, in the study of Najafzadeh et al. it has been identified that peripheral lymphocytes from patients with cancers (malignant melanoma and colorectal cancer) or their precancerous states were more sensitive to a generic mutagen than lymphocytes from healthy individuals [22]. The study of Schmezer et al. did not detect any significant difference between the levels of baseline DNA damage in lymphocytes of lung cancer patients and healthy individuals but found an increased sensitivity of lymphocytes to bleomycin and decreased DNA repair capacity in cancer patients [20]. The deficient DNA repair in lymphocytes of lung, head and neck cancer patients also has been shown by other researchers [4, 5, 8, 9]. It should be mentioned that the concentration of bleomycin (20 μg/ml) recommended by Schmezer et al., which we also used in our study, caused DNA damage at saturation level of the assay. Therefore, we consider that in further research it is more appropriate to use lower concentration of bleomycin, thus increasing the accuracy of measurements of bleomycin sensitivity and DNA repair.

There is no consensus on the causes of genetic instability in lymphocytes of cancer patients. Probably, genome instability in lymphocytes is the result of the influence of reactive oxygen species, increased levels of which were detected in blood of cancer patients [23, 24]. In this way, oxidative stress is observed in breast, lung and colon cancer patients [25–27]. In addition, DNA damage in lymphocytes may also be influenced by tumor-associated factors [12]. Furthermore, reduced repair may lead to an increase in the steady state level of DNA damage.

One study reported that family history of cancer did not have a significant effect on the level of DNA damage in lymphocytes [12]. We also did not find any correlation between a family history of cancer and DNA damage in lymphocytes of EC patients. However, we indeed observed lower DNA repair capacity in lymphocytes of EC patients with a family history of cancer. Perhaps altered DNA repair may be caused by inherited genetic defects and differences in DNA repair capacity reflect individual genetic background leading to different level of genetic instability [5].

We suggest that the comet assay with the use of bleomycin as genotoxic agent is an acceptable way for identifying EC patients with a high level of genome instability. In addition, this assay might be a necessary step for determination of individuals at high risk for cancer among EC patients’ relatives. Further research on genome instability in lymphocytes of cancer patients is needed to investigate its significance for cancer prevention and to identify how well these surrogate cells reflect events at the level of the target tumor tissue.

## Conclusions

The results of our study confirm the importance of measuring the individual capacity to repair DNA damage in EC patients especially in those with a family history of cancer. In the present study, the DNA repair efficiency significantly differs from controls to EC patients and from EC patients with a family history of cancer to those with sporadic cancer. We assume that reduced DNA repair efficiency in peripheral blood lymphocytes of EC patients may reflect the cancer predisposition.

## Abbreviations

EC: Endometrial cancer
PBLs: Peripheral blood lymphocytes.
